# Structure and Chemical Composition of Ion-Synthesized Gallium Oxide Nanocrystals in Dielectric Matrices

**DOI:** 10.3390/nano13101658

**Published:** 2023-05-17

**Authors:** Dmitry S. Korolev, Ruslan N. Kriukov, Kristina S. Matyunina, Alena A. Nikolskaya, Alexey I. Belov, Alexey N. Mikhaylov, Artem A. Sushkov, Dmitry A. Pavlov, David I. Tetelbaum

**Affiliations:** 1 Research Institute of Physics and Technology, Lobachevsky State University of Nizhny Novgorod, 603022 Nizhny Novgorod, Russia; kriukov.ruslan@yandex.ru (R.N.K.); nikolskaya@nifti.unn.ru (A.A.N.); belov@nifti.unn.ru (A.I.B.); mian@nifti.unn.ru (A.N.M.); sushkov@phys.unn.ru (A.A.S.); tetelbaum@phys.unn.ru (D.I.T.); 2 Department of Physics, Lobachevsky State University of Nizhny Novgorod, 603022 Nizhny Novgorod, Russia; matyunina.ks@gmail.com (K.S.M.); pavlov@unn.ru (D.A.P.)

**Keywords:** gallium oxide, nanocrystals, ion-beam synthesis, ion implantation, thermal annealing, X-ray photoelectron spectroscopy, transmission electron microscopy

## Abstract

The ion-beam synthesis of Ga_2_O_3_ nanocrystals in dielectric matrices on silicon is a novel and promising way for creating nanomaterials based on gallium oxide. This research studies the regularities of changes, depending on the synthesis regimes used, in the chemical composition of ion-implanted SiO_2_/Si and Al_2_O_3_/Si samples. It has been shown that the formation of Ga-O chemical bonds occurs even in the absence of thermal annealing. We also found the conditions of ion irradiation and annealing at which the content of oxidized gallium in the stochiometric state of Ga_2_O_3_ exceeds 90%. For this structure, the formation of Ga_2_O_3_ nanocrystalline inclusions was confirmed by transmission electron microscopy.

## 1. Introduction

Current microelectronics technologies that use silicon as the basic material can no longer meet the ever-increasing requirements for electronic devices. Current trends in the development of electronics suggest the use of wide bandgap semiconductors, such as SiC, GaN, and especially Ga_2_O_3_, as the main materials for new-generation devices. However, the creation of these devices on the basis of silicon is difficult or fundamentally impossible [1].

Gallium oxide (Ga_2_O_3_) occupies a leading position in terms of promising applications among wide bandgap semiconductors for a number of vital fields: power electronics, the detection of hard ultraviolet radiation and hazardous gases, high-power computing, nuclear and space industries, medicine, ecology, etc. [2,3]. Ga_2_O_3_ has an ultrawide bandgap (~4.5–5.3 eV depending on the polytype) and high breakdown voltage associated with it. It is also radiation and chemically resistant, among other important features. There are five well-known polytypes of gallium oxide: α (rhombohedral)-, β (monoclinic)-, ε(κ) (orthorhombic)-, δ (cubic)-, and γ (defective spinel)-phase Ga_2_O_3_, the formation of which strongly depend on the synthesis conditions [4,5,6]. Each of the polytypes has unique structural and physicochemical properties that can be used for various applications [7,8,9,10]. For example, β-phase is thermodynamically stable at room temperature and at atmospheric pressure; α-phase has the widest bandgap compared to other polytypes (~5.3 eV); ε(κ)-phase has spontaneous polarization; γ-phase has good catalytic properties and, when doped with Mn atoms, can be used in spintronics; and δ-phase is used in UV detectors, power diodes, etc.

The research and development of Ga_2_O_3_ is experiencing a period of rapid progress [11]. However, the widespread commercial use of gallium oxide is hampered by insufficient fundamental knowledge about the properties of its various polytypes, as well as by the poor compatibility of the Ga_2_O_3_ synthesis and modification methods with traditional microelectronic technologies. Despite the existence of single-crystal substrate growth technologies (including large diameters), the abovementioned methods remain quite expensive, and thin-film growth technologies (including epitaxial ones) have a large number of unsolved problems that limit their practical application [3].

One promising approach to the creation of Ga_2_O_3_-based materials can be the use of nanostructured materials; in particular, the creation of nanosized gallium oxide inclusions of various polymorphic modifications [6]. The use of nanoscale Ga_2_O_3_ inclusions could considerably expand the application of this material, particularly for heterogeneous catalysis problems [12], as well as for efficient luminescent materials in the UV spectral range [13]. In addition, nanostructured gallium oxide allows for more flexibility in changing the properties of the structures compared to the use of single-crystal substrates and thin-film magnetron or epitaxial structures. However, at the moment, the primary way of creating Ga_2_O_3_ nanostructures requires the application of various chemical methods (a detailed review of synthesis techniques is given in [14]), which are almost incompatible with conventional complementary metal–oxide–semiconductor (CMOS) technology.

One of the promising methods for creating structures with gallium oxide nanocrystals is the method of ion synthesizing Ga_2_O_3_ nanocrystals (nc-Ga_2_O_3_) in solid matrices. Ion implantation—a foundation of this method—has been one of the main operations of CMOS technology for many years, and it has already proved its effectiveness for materials based on gallium oxide [15]. The ion synthesis technique of nanocrystals has been developed for a number of semiconductor materials, such as Si [16], SiC [17], ZnO [18], GaN [19], and In_2_O_3_ [20]. However, the creation of Ga_2_O_3_ nanocrystals achieved by using this method has not been previously reported, except for our recent works [21,22].

Previously, we demonstrated the possibility of ion-beam syntheses of Ga_2_O_3_ nanocrystals in dielectric matrices, showed the promise of using this method to create “solar-blind” photodetectors [21], and studied their structural and light-emitting properties [22]. However, the change in the chemical composition of the synthesized structures as a result of post-implantation annealing—which plays a determining role in the formation of Ga_2_O_3_ nanocrystalline inclusions, as well as the influence of the post-implantation annealing atmosphere—have not been studied yet.

In this paper, the regularities of the physical and chemical processes occurring during the ion synthesis of Ga_2_O_3_ nanocrystalline inclusions in the dielectric matrices of SiO_2_ and Al_2_O_3_ on silicon are considered. The article also features data on the change in the structural and chemical composition, depending on the conditions of post-implantation thermal treatment, of the synthesized samples.

## 2. Materials and Methods

**Figure 1:**
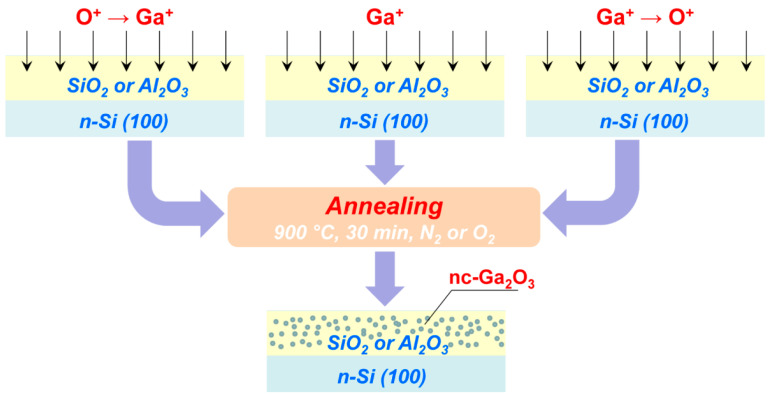
Scheme of the used variants for the ion-beam synthesis of nc-Ga_2_O_3_.

**Figure 2:**
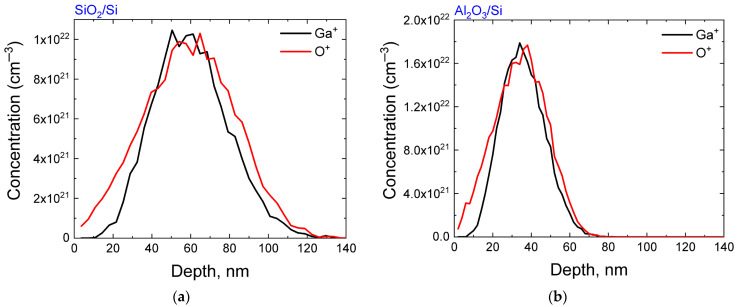
SRIM-calculated distribution of implanted gallium and oxygen ions: (**a**) for SiO_2_/Si structures; (**b**) for Al_2_O_3_/Si structures.

Silicon n-Si (100) wafers were used as the initial samples, on which SiO_2_ and Al_2_O_3_ films were deposited by electron-beam deposition. The thickness of the films was ~350 nm and ~200 nm, respectively. Ion-beam synthesis includes two consecutive stages: irradiation with ions of phase-forming elements and post-implantation annealing (Figure 1). In the first stage of synthesis, Ga ions (80 keV, 5 × 10^16^ cm^−2^) and O_2_ ions (45 keV, 3 × 10^16^ cm^−2^) were implanted with variation in the irradiation order. The implantation of only gallium ions was also used since it was expected that oxygen from the oxide matrix would be involved in the creation of the Ga-O chemical bonds, which in turn form Ga_2_O_3_ nanocrystalline inclusions. The implantation of molecular oxygen ions instead of atomic ones was used to reduce the total irradiation time. When colliding with the sample surface, the molecular O_2_^+^ ions decomposed to form two ions with half the energy. The irradiation regimes were selected based on the condition of maximum coincidence of the ion distribution profiles, as calculated using SRIM-2013 software (www.srim.org, accessed on 10 April 2023). In the calculations, the density of SiO_2_ and Al_2_O_3_ was taken as 2.32 and 3.98 g/cm^3^, respectively. The displacement energies were 15, 25, and 28 eV for Si, Al, and O atoms, respectively. The maximum of the implanted atoms (R*_p_*) was at a depth of ~60 nm for SiO_2_/Si structures and ~35 nm for Al_2_O_3_/Si structures (Figure 2). At the second stage of ion synthesis, a single annealing procedure was carried out in a tube furnace at 900 °C (30 min) in N_2_ and O_2_ atmospheres.

The composition and chemical state of the initial samples were investigated by X-ray photoelectron spectroscopy (XPS), and implemented on the basis of an Omicron MultiprobeRM (Omicron, Germany) ultrahigh-vacuum complex. O 1*s*, C 1*s*, Ga 2*p_3/_*_2_, and Si 2*s* photoelectronic lines were registered. The acquisition area was ~3 mm^2^. Spectra were taken at the transmittance energy of the analyzer of 50 eV and an energy discretization of 0.2 eV/step. Ar^+^ ion etching with an energy of 1 keV and an etch region diameter of 20 mm was used to obtain chemical element depth distribution profiles. The angle of incidence of the ion beam to the sample surface was 45°. The experimental procedure and data analysis are described in detail in [23].

The structure of the samples was investigated by a transmission electron microscopy (TEM) of the cross-section on a JEM-2100F (JEOL, Tokyo, Japan) microscope using a 601.07000 TEM Specimen Preparation Kit (Gatan, Inc., Pleasanton, CA, USA) for preparation, which was conducted according to the Gatan method (USA).

## 3. Results and Discussion

### 3.1. Composition of as-Implanted SiO_2_/Si and Al_2_O_3_/Si Samples

**Figure 3:**
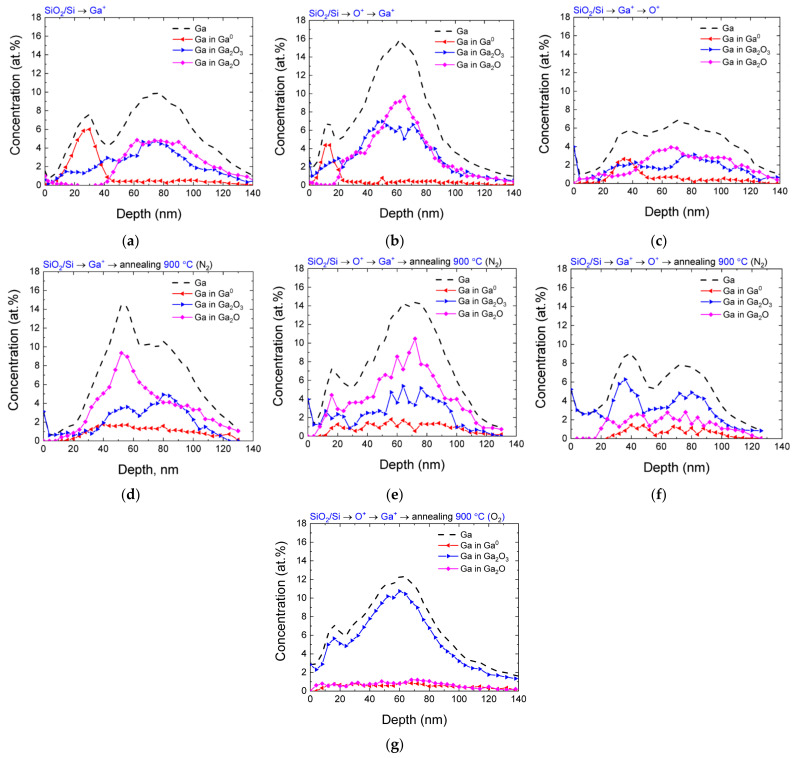
Distribution profile of gallium (dashed line), as well as gallium in various chemical states (Ga^0^, Ga_2_O_3_, Ga_2_O, colored lines), over depth for different variants of the implantation order for the SiO_2_/Si samples: (**a**–**c**) before annealing; (**d**–**f**) after annealing at a temperature of 900 °C in an N_2_ atmosphere; and (**g**) for the O^+^ → Ga^+^ implantation order after annealing at 900 °C in an O_2_ atmosphere.

The study of the chemical composition of the implanted samples before and after annealing was carried out by the XPS method, which makes it possible to implement elemental analysis by taking into account the shifts of the photoelectron lines that are caused by the chemical bonds of atoms with their environment. In our previous work [22], the distribution profiles of implanted gallium and oxygen atoms in SiO_2_/Si samples that were not subjected to post-implantation annealing were investigated. It was established that, even in the absence of annealing, all samples contain not only gallium in the elemental state, but also oxidized gallium in the stoichiometric Ga_2_O_3_ and the oxygen-deficient Ga_2_O states. The distribution profiles of gallium (Figure 3) indicated the presence of two maxima. The first one predominantly contained gallium in the elemental state and was closer to the surface. The second maximum was located at depths that were close to the maximum of the implanted impurity distribution and deeper. Moreover, almost all implanted gallium at this depth was in the oxidized state. Additionally, of interest is the fact that the lines associated with the presence of Ga-O bonds were observed even in the absence of additional implantation by oxygen ions. This indicated the participation of oxygen from the SiO_2_ matrix in the oxidation process.

As can be seen in Figure 3, the order of implantation of Ga^+^ and O^+^ ions had a significant effect on the concentration and profile of gallium with different oxidation states. Indeed, the implantation of oxygen prior to the implantation of gallium led to a higher total concentration of the latter, as well as to a higher degree of its oxidation; meanwhile, the implantation of O^+^ after Ga^+^ does not contribute to the oxidation of previously implanted gallium, and it also causes its significant desorption.

This can be explained as follows. It is known that ion implantation is characterized by the appearance of so-called “thermal spikes” during the introduction of ions [24]. This phenomenon is more pronounced for heavy ions, which form dense displacement cascades. Gallium is one such ion. Apparently, during its implantation in SiO_2_, local heating in the region of ion tracks promotes the reaction of Ga with matrix oxygen and the formation of gallium oxides. Local heating is most pronounced in the region close to the average projected range *R*_p_(Ga), where the energy density released in elastic collisions is the highest, hence the local temperature is higher; on the other hand, the Ga located closer to the surface is partially preserved in the elemental state and forms the near-surface maximum observed in Figure 3a. If the matrix is additionally enriched with oxygen by irradiation with O^+^ before Ga^+^ implantation, the degree of Ga oxidation becomes even higher. Moreover, since the unbound gallium can undergo radiation-stimulated desorption, the pre-implantation of O^+^ weakens it due to a more complete binding of gallium to oxygen in the SiO_2_ matrix. Oxygen ions, being lighter, create less dense cascades compared to Ga^+^ and, for them, the effect of “thermal spikes” is expressed to a much lesser extent; therefore, O^+^ implantation after Ga^+^ implantation does not contribute to the oxidation of Ga, and it even leads to an additional radiation-accelerated desorption (apparently due to the knocking of Ga out of the lattice sites and its transfer to a more mobile interstitial state).

**Figure 4:**
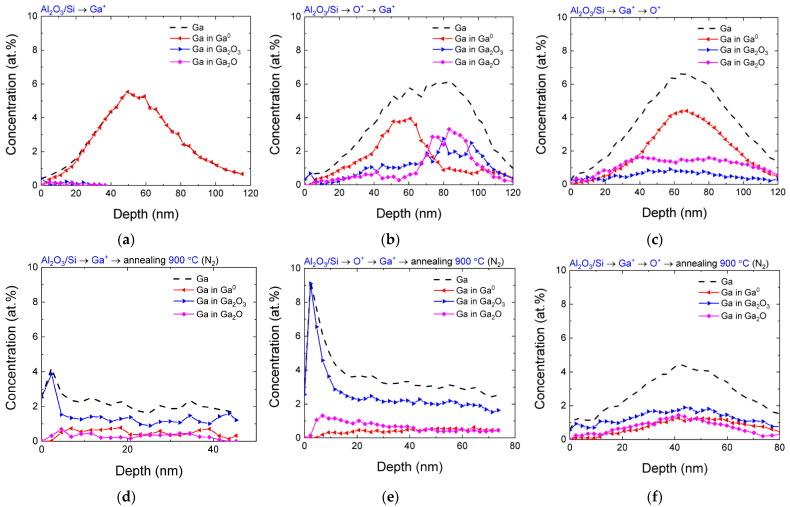
Distribution profile of gallium (dashed line), as well as gallium in various chemical states (Ga^0^, Ga_2_O_3_, Ga_2_O, colored lines), over depth for different variants of the implantation order for the Al_2_O_3_/Si samples: (**a**–**c**) before annealing; (**d**–**f**) after annealing at 900 °C in a N_2_ atmosphere.

For the implanted Al_2_O_3_/Si samples, the situation is different (Figure 4). The main difference from the case of implantation into the SiO_2_ matrix is the lower concentration of gallium at the same implantation dose. Another important peculiarity is the almost complete absence of oxidized gallium in the case of only Ga^+^ implantation without additional irradiation with oxygen. This indicates that the oxygen from the matrix hardly participates in the oxidation of implanted Ga.

In the case of oxygen implantation prior to gallium, two maxima appear on the depth distribution profiles of gallium: the first one mainly consists of gallium in the elemental state, while the second one is due to the presence of oxidized gallium in the Ga_2_O and Ga_2_O_3_ states. A similar situation was also observed in the SiO_2_/Si matrix; however, there was a noticeable shift in the elemental gallium profile that was closer to the surface. In addition, a common feature of the distribution profiles in the Al_2_O_3_/Si matrix is a significantly greater impurity depth compared to that calculated using the SRIM program (Figure 1). In the case of oxygen implantation after gallium ions, the total gallium profile is close to the case of O^+^ → Ga^+^ implantation, but a bimodal peak structure was not observed in this case. In this case, the gallium atoms were predominantly in the elemental state, and only a small part of the implanted atoms (~1–2 at.%) was in the oxidized state with different degrees of oxidation. This is additional evidence that, in the absence of annealing, the participation of oxygen from the matrix for oxidation is hindered. This process also requires the matrix, into which gallium atoms are implanted, to be initially oversaturated with oxygen.

A separate consideration of the peculiarity of the Ga depth distribution in the Al_2_O_3_/Si matrix, which is associated with a significantly greater impurity depth compared to the calculated one, shows that this effect is observed for all variants of implantation. However, it has its peculiarities in each case. In the case when only gallium atoms are implanted, the shift of the maximum is ~10 nm. Upon implantation of Ga^+^ → O^+^, this shift increases. In the case of oxygen implantation before gallium ions, the first maximum approximately coincides in position with the previous ones, and the second maximum relates mainly to gallium bound with oxygen, which is located at much greater depths. The nature of this phenomenon is associated with the effect of radiation-accelerated diffusion. The fact that the increase in the depth of gallium distribution is more pronounced in the case of additional oxygen implantation can be explained as follows. If it is assumed that gallium diffuses by the interstitial mechanism, then the presence of excess oxygen vacancies in the irradiated film leads to their capture of diffusing gallium atoms, which slows down diffusion. In the case of preliminary irradiation with oxygen ions, some of the oxygen vacancies are filled; as a result, the diffusion flux of gallium atoms increases. The preferential capture of gallium atoms by oxygen vacancies in the absence of preliminary irradiation with oxygen ions is also confirmed by the low concentration of Ga-O bonds (Figure 4a) since the gallium atom that has fallen into the vacancy position is surrounded by other oxygen atoms and does not form other bonds with oxygen. In the case of the reverse order of implantation, the effect of the shift toward greater depths is less pronounced.

### 3.2. Composition of Implanted SiO_2_/Si and Al_2_O_3_/Si Samples after Annealing

The distribution profiles of implanted gallium atoms in the samples after annealing at 900 °C in a nitrogen atmosphere are presented on Figure 3d–f. A common feature for the SiO_2_/Si samples implanted in different regimes is a significant decrease in the concentration of gallium atoms in the elemental state. At the same time, no noticeable decrease in the total concentration of gallium was observed. For a sample implanted only with Ga ions, the near-surface peak related to elemental gallium disappears after annealing. Together with the process of oxidation of gallium, its diffusion toward greater depths is observed. In this case, oxidized gallium is in an oxygen-deficient Ga_2_O state and its concentration in the stoichiometric composition practically does not change after annealing. This confirms the hypothesis that there is a critical concentration of oxygen atoms in the matrix, which can participate in the formation of Ga-O bonds. After this formation is complete, the process of oxidation of the implanted gallium significantly slows down. In the case of Ga^+^ → O^+^ implantation, the redistribution of gallium and its insignificant diffusion toward the surface are also observed. Annealing of these samples leads to the additional oxidation of both metallic gallium and gallium in the oxygen-deficient state, which contributes to an increase in the fraction of gallium in the stoichiometric Ga_2_O_3_ state.

Finally, for the O^+^ → Ga^+^ implantation order, the total distribution profile of gallium atoms remains almost unchanged after annealing. The oxidation of gallium, which is in the elemental state before annealing, was observed. For this order of implantation, there was a slight decrease in the concentration of gallium in the stoichiometric Ga_2_O_3_ state with a simultaneous increase in the concentration of Ga-O bonds with a lack of oxygen. Based on the presented data, it can be concluded that the final distribution of gallium atoms in various chemical states is determined not only by the diffusion of gallium (both radiation-stimulated and thermal diffusion during annealing), but also by the possible diffusion of oxygen atoms from this region. This assumption is supported by the following facts. First, according to the results obtained for the case of implantation of Ga ions only, it can be noted that there is a limited number of oxygen atoms from the matrix that can participate in the oxidation process. Second, it can be expected that the oxidation of gallium in the elemental state as a result of annealing can be determined not only by the processes of chemical interaction at elevated temperatures, but also by the influx of oxygen atoms into the near-surface region from the depth.

Since a significant decrease in the concentration of oxidized gallium in the stoichiometric state of Ga_2_O_3_ was observed for this variant of ion synthesis, an attempt to increase it was made by annealing the samples in an oxygen atmosphere, while all other annealing conditions (temperature and duration) remained the same. The study of the chemical composition revealed an interesting effect—namely, the almost complete oxidation of implanted gallium to the stoichiometric Ga_2_O_3_ state (Figure 3g). This fact additionally supports the conclusion that, for a more efficient oxidation of gallium, it is necessary to provide such conditions that ensure the sufficient oxygen content in the implanted region for obtaining the stoichiometric composition of Ga_2_O_3_. In our case, during the implantation of Ga^+^ and O^+^, the concentrations of oxygen and gallium atoms were equal, and the additional participation of oxygen from the matrix was insufficient for obtaining a stoichiometric composition. This fact requires additional verification and will be investigated in the framework of further research.

The bimodal distribution of gallium, which is observed in almost all distribution profiles, especially for samples after annealing, is noteworthy. It can be assumed that the formation of an additional peak, which is closer to the surface compared to the calculated maximum of the impurity distribution, is associated with the formation of radiation defects, and the distribution maximum of which is usually located closer to the surface. The sink of gallium atoms into the defect-enriched region can occur due to radiation-stimulated diffusion, which leads to the appearance of an additional peak in the gallium concentration profile.

The distribution profiles of Ga atoms in the implanted Al_2_O_3_/Si samples after annealing are presented on Figure 4d–f. A common feature of all the samples was the loss of implanted gallium as a result of annealing. This effect was the most pronounced for the samples implanted only with Ga^+^, in which an almost uniform distribution of gallium with a concentration of ~2 at.% was observed. For a sample implanted in the O^+^ → Ga^+^ order, a decrease in the total concentration of implanted gallium was also observed with its simultaneous diffusion to the surface. It should be noted that, despite the decrease in the total concentration, gallium in the samples after annealing predominantly remained in the fully oxidized Ga_2_O_3_ state. Finally, for the Ga^+^ → O^+^ implantation order, the impurity loss was less pronounced. This may be due to the formation of stable complexes containing gallium and oxygen atoms since, compared to the reverse order of ion implantation, this profile does not split into elemental Ga and gallium in the oxidized state. In any case, after annealing, the total concentration of gallium in the oxidized state for all the used variants of ion synthesis was too low for the efficient formation of an array of Ga_2_O_3_ nanoinclusions in the Al_2_O_3_/Si matrix.

### 3.3. Structural Properties of Implanted SiO_2_/Si Samples after Annealing in the Oxygen Atmosphere

**Figure 5:**
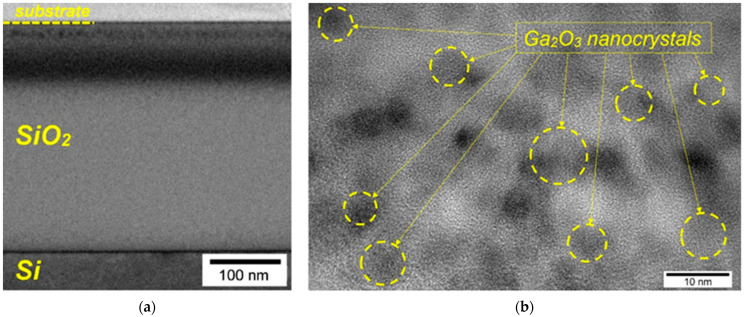
(**a**) Cross-sectional image of the SiO_2_/Si samples: the O^+^ + Ga^+^ structure after the final annealing at 900 °C in an O_2_ atmosphere; (**b**) high-resolution TEM image of the same sample, on which the synthesized nc-Ga_2_O_3_ are highlighted.

According to the XPS data, the most effective in terms of obtaining the maximum concentration of oxidized gallium in the state of stoichiometric Ga_2_O_3_ was the SiO_2_/Si sample that was irradiated first with O ions and then with Ga ions after annealing at a 900 °C in an oxygen atmosphere. In this case, the efficiency of the formation of chemical bonds corresponding to the stoichiometric Ga_2_O_3_ composition exceeded 90%. To study the structural properties of this sample, the method of transmission electron microscopy, including a high resolution, was used. Figure 5 shows the cross-sectional patterns of the SiO_2_/Si samples: the O^+^ + Ga^+^ structure after annealing in an oxygen atmosphere at 900 °C. In the overview of a bright-field TEM image (Figure 5a), the formation of two layers of dark contrast was observed. This correlates with the XPS data, according to which the gallium distribution profile exhibited a weak peak at depths of ~20 nm, and a broad intense peak at a depth of ~60 nm.

The high-resolution image obtained in the region of darker contrast (Figure 5b) shows the formation of a large number of spherical nanoinclusions with sizes of ~4–9 nm, some of which are indicated in the figure. For the inclusions with atomic planes observed in the TEM images, processing was carried out by measuring the interplanar distances and interpreting the diffraction patterns obtained by the Fourier transform method. An analysis of the obtained data shows that Ga_2_O_3_ nanoinclusions are formed in two different crystalline phases, β-Ga_2_O_3_ and γ-Ga_2_O_3_. This partially agrees with the previously obtained data on the ion synthesis of the nc-Ga_2_O_3_ obtained by annealing in a nitrogen atmosphere, for which the formation of β-phase nanocrystals was found [22]. One of the possible explanations for the observed difference can be the following. As was assumed earlier [22], during the ion synthesis of Ga_2_O_3_ nanocrystals, chemical bonds between gallium and oxygen (Ga_x_O_y_ complexes) were formed after irradiation. These bonds serve as the centers for the nucleation of a new phase. During annealing, the process of nanocrystal formation goes through several stages. First, small non-phase inclusions containing Ga and O atoms are formed as nuclei, which can later be enlarged due to the addition of new atoms. With an increase in annealing temperature, the formation of nanoinclusions in the metastable γ-Ga_2_O_3_ phase that contain a large number of structural defects is possible. A further increase in annealing temperature and time leads to the preferential transition of nanoinclusions to the stable β-phase. However, in the annealing conditions used in this work (oxygen-containing atmosphere), this transition did not complete; therefore, the presence of the nanocrystals of both phases was observed in the samples. Comparing these data with the previously obtained results for the case of step-by-step annealing in a nitrogen atmosphere [22], it can be assumed that, along with the influence of the annealing atmosphere, the “history” of heat treatments can also play a certain role. The mechanism of phase transitions in nc-Ga_2_O_3_ under various heat treatment conditions requires separate investigations.

## 4. Conclusions

The regularities of changes in the chemical composition of the SiO_2_/Si and Al_2_O_3_/Si samples during the ion synthesis of Ga_2_O_3_ nanocrystalline inclusions were studied depending on the conditions of irradiation with Ga and O ions and the subsequent heat treatment. It was shown that the formation of Ga-O bonds occurs even in the absence of post-implantation annealing, and their formation significantly depends on the order of ion irradiation. For the Al_2_O_3_/Si matrix, the loss of implanted gallium was observed as early as at the irradiation stage, and subsequent heat treatment led to a further decrease in the Ga concentration. Therefore, the implantation and annealing conditions used made the ion synthesis of Ga_2_O_3_ nanocrystals in the Al_2_O_3_/Si matrix very difficult.

Annealing implanted SiO_2_/Si samples leads to a decrease in the fraction of gallium in the elemental state, with a simultaneous increase in the concentration of oxidized gallium in the oxygen-deficient states (Ga_2_O) and in the stoichiometric state (Ga_2_O_3_). The use of oxygen instead of nitrogen in the annealing atmosphere leads to an increase in the content of gallium in the stoichiometric state of Ga_2_O_3_ above 90%. The study of the structure of the sample obtained, by transmission electron microscopy, under such synthesis conditions revealed the formation of the nanocrystalline inclusions of γ- and β-Ga_2_O_3_, with an average size of 4–9 nm.

Thus, this study demonstrates the possibility of controlling the chemical composition and properties of the ion-synthesized Ga_2_O_3_ nanocrystalline inclusions by varying the parameters of synthesis. This feature of the ion synthesis method makes it possible to create structures with repeatable and controllable properties using ion implantation and thermal annealing processes that are fully compatible with traditional electronic technology. Further development of the technology for creating gallium oxide nanocrystals using the method of ion synthesis in silicon-compatible dielectric matrices opens up prospects for the wide application of this technique in the fabrication of a new generation of electronic devices based on gallium oxide.

## Data Availability

The data presented in this study are available on request from the corresponding authors.
